# Neonatal Presentation of a Case of Carbonic Anhydrase VA Deficiency

**DOI:** 10.7759/cureus.90845

**Published:** 2025-08-23

**Authors:** Saja Baheer Abdulwahhab, Amna Ahmed, Tawfeg Ben Omran

**Affiliations:** 1 Department of Genetics, Sidra Medicine, Doha, QAT; 2 College of Health Sciences, Qatar University, Doha, QAT; 3 Department of Medical Genetics, Hamad Medical Corporation, Doha, QAT; 4 College of Medicine, Weill Cornell Medicine-Qatar, Doha, QAT

**Keywords:** carbonic anhydrase va deficiency, ca-va deficiency, gene, hyperammonemia, metabolic acidosis, neonate

## Abstract

Carbonic anhydrase VA (CA-VA) deficiency is a rare mitochondrial disorder affecting bicarbonate-dependent metabolic pathways. It commonly presents in neonates with hyperammonemia, lactic acidosis, and metabolic crisis, often mimicking more prevalent urea cycle and organic acid disorders. This rare metabolic disorder has been reported across different populations, with cases documented worldwide. We report the case of a three-day-old full-term Indian female neonate presenting with lethargy and poor feeding. Initial labs revealed hyperammonemia, severe lactic acidosis, hypoglycemia, and ketosis. Despite ammonia scavenger therapy, hyperammonemia persisted, necessitating continuous renal replacement therapy. Metabolic workup suggested mitochondrial dysfunction. Genetic testing identified a homozygous exon 6 deletion in the CA5A gene, confirming CA-VA deficiency. She was discharged on carglumic acid and supplements, with clinical and biochemical improvement. CA-VA deficiency should be included in the differential diagnosis of neonatal metabolic decompensation, particularly in South Asian infants. Early intervention can lead to favorable outcomes, underscoring the importance of prompt metabolic and genetic evaluation.

## Introduction

Carbonic anhydrase VA (CA-VA) deficiency is a rare inborn error of metabolism resulting from autosomal recessive mutations in the CA5A gene, which leads to a deficiency of the mitochondrial enzyme CA-VA [1]. Clinically, the condition typically presents in the neonatal or early infantile period with acute metabolic crises characterized by metabolic acidosis and hyperammonemia [1]. A significant proportion of reported cases have been observed in individuals of South Asian origin, suggesting a possible genetic predisposition within this population [2]. Management of CA-VA deficiency involves supportive care with intravenous fluids, correction of acidosis, and targeted treatment of hyperammonemia, with carglumic acid demonstrating rapid ammonia normalization in most cases [3]. Despite the severity of initial presentations, the long-term prognosis is generally favorable, with the majority of affected individuals achieving normal psychomotor development, although some may experience mild learning difficulties or motor delays [3].

## Case presentation

A three-day-old full-term Indian female neonate, born after an uneventful pregnancy and with normal neonatal screening, presented to the emergency department with lethargy and poor feeding. Initially suspected to have neonatal sepsis, she was admitted and evaluated with laboratory investigations that revealed several metabolic abnormalities, prompting further consideration of an inborn error of metabolism. The combination of clinical deterioration and biochemical profile raised concern for a possible mitochondrial disorder.

Initial laboratory findings (Table 1) showed severe metabolic acidosis (pH 7.21; HCO₃⁻ 9 mmol/L), markedly elevated lactate (14 mmol/L), a high anion gap (29), hypoglycemia (2.3 mmol/L), and profound hyperammonemia (553 μmol/L). Ketosis was also evident on urinalysis.

**Table 1:** Initial Laboratory Investigations

Parameter	Result	Reference Range	Units
pH	7.21	7.35–7.45	—
Bicarbonate (HCO₃⁻)	9	22–28	mmol/L
Lactate	14	0.5–2.2	mmol/L
Anion Gap	29	8–16	—
Glucose	2.3	2.8–6.1	mmol/L
Ammonia	553	<50	μmol/L

Clinical course

The infant developed progressive encephalopathy with rapid worsening of metabolic parameters. Given the high ammonia level, initial management prioritized metabolic stabilization. Intravenous fluids containing dextrose were administered to correct hypoglycemia and suppress protein catabolism. Nitrogen-scavenging agents, sodium benzoate and sodium phenylacetate, were initiated promptly to promote alternative nitrogen excretion pathways. These interventions led to a temporary decrease in serum ammonia; however, levels rebounded within hours, indicating an inadequate response. Due to ongoing neurological compromise and worsening metabolic status, continuous renal replacement therapy (CRRT) was started, which successfully normalized serum ammonia within 24 hours. The infant’s neurological condition improved significantly post CRRT, allowing extubation within 48 hours. This case highlights the need for early escalation to extracorporeal ammonia removal when pharmacological measures are insufficient.

Differential diagnosis and workup 

Given the severity of hyperammonemia, urea cycle disorders (UCDs) were initially considered. However, plasma amino acid profiling revealed elevated alanine, glutamine, and proline, without accumulation of typical urea cycle intermediates. Additionally, urinary orotic acid was not elevated, arguing against common UCDs such as ornithine transcarbamylase deficiency. Organic acidemias were also considered, but urinary organic acid analysis showed elevated lactate, ketones, and pyroglutamic acid without classical patterns seen in disorders like methylmalonic, propionic, or isovaleric acidemia.

Advanced metabolic workup

Urinary organic acid analysis demonstrated elevated lactic acid and multiple ketone bodies (3-hydroxybutyrate, 2-hydroxybutyrate, acetoacetate, and 3-hydroxyisovaleric acid), along with moderate pyruvate elevation and increased pyroglutamic acid (5-oxoproline). No classical organic aciduria pattern was identified. Plasma amino acid analysis confirmed markedly elevated alanine (2,781.6 μmol/L; reference range: 114-380 μmol/L), glutamine (1,802.7 μmol/L; reference range: 451-1,113 μmol/L), and proline (754.8 μmol/L; reference range: 127-292 μmol/L), all consistent with mitochondrial dysfunction. Carnitine levels were within the normal range, and the acylcarnitine profile showed only mild, nonspecific elevations.

Genetic testing and diagnosis

Given the constellation of biochemical findings and poor initial response to standard therapy, whole exome sequencing was performed. This revealed a pathogenic deletion of exon 6 in the CA5A gene, confirming the diagnosis of CA-VA deficiency, a rare mitochondrial disorder that impairs intra-mitochondrial bicarbonate supply and urea cycle activation.

Outcome

Following diagnosis, targeted therapy with carglumic acid (100 mg/kg/day) was initiated to activate carbamoyl phosphate synthetase I and enhance ammonia clearance. The patient also received mitochondrial cofactors (biotin, riboflavin, and coenzyme Q10) to support mitochondrial function. Exclusive breastfeeding was continued with careful metabolic monitoring. The infant showed sustained clinical improvement and was discharged in stable condition on day 20 of life. At five weeks of age, she remained clinically well with age-appropriate developmental milestones and normal biochemical parameters on follow-up.

This case emphasizes the importance of a systematic and timely approach to neonatal hyperammonemia, especially when initial therapy fails. Detailed knowledge of escalation pathways, including the early use of CRRT and disease-specific therapies like carglumic acid, can significantly improve outcomes in rare but treatable conditions such as CA-VA deficiency. Recognizing the triad of hyperammonemia, lactic acidosis, and ketosis, in combination with a structured diagnostic approach, is crucial for early identification and management of this disorder in clinical practice.

## Discussion

Neonatal hyperammonemia accompanied by lactic acidosis presents a broad differential diagnosis, with UCDs and organic acidurias being among the most frequently encountered causes. One increasingly recognized, though rare, condition that can closely mimic these is CA-VA deficiency. This mitochondrial disorder shares several clinical features with UCDs, particularly carbamoylphosphate synthetase 1 (CPS1) deficiency and N-acetylglutamate synthase (NAGS) deficiency, most notably the presence of hyperammonemia. However, CA-VA deficiency is distinguished by its characteristic metabolic profile, which includes not only hyperammonemia but also metabolic acidosis, lactic acidosis, and ketosis. These findings reflect the impaired mitochondrial bicarbonate metabolism, which is essential for activating multiple carboxylase enzymes. Impairment of mitochondrial carbonic anhydrase activity disrupts these processes, resulting in secondary metabolic decompensation [3,4].

In contrast to UCDs, which often present with isolated hyperammonemia and minimal acid-base imbalance, CA-VA deficiency is marked by a more complex biochemical signature. For instance, CPS1 or NAGS deficiency typically lacks the pronounced lactic acidosis and ketosis observed in CA-VA deficiency. In our patient, the simultaneous presence of hyperammonemia, lactic acidosis, and elevated ketone bodies pointed strongly toward mitochondrial dysfunction rather than a primary urea cycle defect. This metabolic profile is consistent with previous case reports, including those by Van Karnebeek et al., which demonstrated that concurrent ketosis and lactic acidosis help differentiate CA-VA deficiency from classic UCDs [5,6].

To aid in clinical interpretation, the key laboratory findings in our patient are presented below in Table 1, which highlights the significant metabolic abnormalities that prompted further diagnostic evaluation.

Further differentiation from organic acidurias such as pyruvate carboxylase deficiency (PCD) is supported by the absence of hallmark metabolites. In PCD, severe lactic acidosis is commonly associated with elevations in methylcitrate and 3-hydroxypropionate, neither of which was detected in our case. Instead, elevated levels of pyroglutamic acid (5-oxoproline) were noted. Pyroglutamic acid, a component of the γ-glutamyl cycle, accumulates when carbonic anhydrase activity is deficient, leading to impaired glutathione recycling and secondary metabolic acidosis. Although not specific to CA-VA deficiency, the presence of pyroglutamic acid in the context of lactic acidosis, hyperammonemia, and ketosis is a significant diagnostic clue. This finding, also reported in previous cases of CA-VA deficiency, adds to the biochemical profile supporting the diagnosis [6,7].

Another point of clinical relevance is the treatment response. Disorders such as multiple carboxylase deficiency (MCD) often improve with biotin supplementation, a feature not seen in CA-VA deficiency. Instead, CA-VA deficiency requires targeted management, focusing on rapid ammonia reduction using nitrogen-scavenging agents and, in more severe cases, renal replacement therapy. In our patient, these strategies were employed effectively. Case reports by Stockdale et al. and Konanki et al. have emphasized the importance of early ammonia control and the role of carglumic acid in improving metabolic outcomes in neonates with CA-VA deficiency [8,9].

Ethnic predisposition is another critical factor in CA-VA deficiency. A higher incidence has been reported among individuals of South Asian descent, particularly those of Indian origin, likely due to a founder mutation in the CA5A gene. This highlights the importance of genetic counseling and population-targeted genetic screening. Our patient, a female infant of Indian heritage, exemplifies this pattern. Studies such as that by Al-Thihli et al. underscore the significance of early diagnosis and counseling in at-risk populations to enable prompt and precise treatment [9].

Clinically, CA-VA deficiency often presents as hyperammonemic encephalopathy in the neonatal period, typically triggered by physiological stressors such as illness or fasting, both of which were evident in our case. Acute management strategies include intravenous ammonia scavengers such as sodium benzoate and sodium phenylacetate, as well as renal replacement therapy when conservative measures are insufficient. In our patient, carglumic acid at a dose of 100 mg/kg/day, functioning as an analog of N-acetylglutamate (NAG), effectively normalized ammonia levels.

Timely recognition and treatment are essential to prevent irreversible neurological injury. While most infants with early intervention demonstrate favorable neurodevelopmental outcomes, mild cognitive or motor delays have been observed. Longitudinal studies and reports by Van Karnebeek et al. and Stockdale et al. note that, despite early metabolic stabilization, some children may exhibit subtle developmental delays, emphasizing the need for long-term follow-up [5,6].

## Conclusions

CA-VA deficiency is a rare but increasingly recognized cause of neonatal hyperammonemia, particularly when accompanied by lactic acidosis and ketosis. This distinct metabolic profile sets it apart from more common inborn errors of metabolism, such as urea cycle defects and organic acidemias, which typically present with either isolated hyperammonemia or specific patterns of organic acid accumulation. Recognizing this differentiation is critical, as CA-VA deficiency can be easily misdiagnosed during the initial stages of a metabolic crisis. Genetic counseling and early diagnosis play a pivotal role in the effective management of CA-VA deficiency, especially in ethnically predisposed populations such as those of South Asian descent, where a founder mutation in the CA5A gene has been identified. In these groups, early genetic screening can lead to prompt diagnosis, enabling timely therapeutic intervention and potentially avoiding severe complications.

Initiating treatment early with ammonia scavengers, including sodium benzoate and sodium phenylacetate, along with carglumic acid, has been shown to result in favorable short-term outcomes by rapidly reducing ammonia levels and preventing neurological deterioration. However, despite early biochemical control, long-term neurodevelopmental monitoring remains essential, as subtle cognitive or motor delays may emerge later in childhood. This case highlights the critical need for heightened clinical awareness and consideration of CA-VA deficiency in the differential diagnosis of neonatal metabolic crises. Early recognition, appropriate laboratory workup, and prompt genetic testing can significantly improve outcomes and guide long-term care in affected infants.
